# Plasma Pyridoxal 5´-Phosphate Level in Children with Intractable and Controlled Epilepsy

**Published:** 2017

**Authors:** Zahra PIRZADEH, Mohammad GHOFRANI, Mohsen MOLLAMOHAMMADI

**Affiliations:** 1 Pediatric Neurology Department, Children Growth Research Center, Qazvin University of Medical Sciences, Qazvin, Iran; 2 Pediatric Neurology Research Center, Shahid Beheshti University of Medical Sciences (SBMU), Tehran, Iran; 3 Pediatric Neurology Excellence Center, Pediatric Neurology Department, Mofid Children Hospital, Faculty of Medicine, Shahid Beheshti University of Medical Sciences (SBMU), Tehran, Iran; 4 Pediatric Neurology, Hazrat Fatemeh Masoumeh Pediatric Hospital,Qom University of Medical Sciences, Qom, Iran

**Keywords:** Pyridoxine Dependent Epilepsy, Intractable Epilepsy, Plasma Pyridoxal Phosphate Level, Children

## Abstract

**Objective:**

Intractable epilepsy is a serious neurologic problem with different etiologies. Decreased levels of pyridoxal phosphate in cerebral spinal fluid of patients with intractable epilepsy due to pyridoxine dependency epilepsy are reported. The aim of this study was to compare plasma pyridoxal 5´-phosphate level in patients with intractable and controlled epilepsy.

**Materials & Methods:**

This cross- sectional analytic study included 66 epileptic children, 33 patients with controlled and 33 patients with intractable epilepsy, after neonatal period up to 15 yr old of age. Thirty-three patients with intractable epilepsy (10- 162 months) and 33 patients with controlled epilepsy (14-173 months) were enrolled. The study was conducted in Pediatric Neurology Clinic of Mofid Children Hospital, Tehran, Iran from January 2010 to December 2010. Patients’ clinical manifestations, laboratory and neuroimaging findings were collected. Non-fasting plasma 5´- pyridoxal phosphate levels of these subjects were assessed by high-pressure liquid chromatography.

**Results:**

Mean plasma 5´- pyridoxal phosphate level (PLP) in patients with controlled epilepsy was 76.78±37.24 (nmol/l) (15.5-232.4). In patients with intractable epilepsy, mean plasma 5´- pyridoxal phosphate was 98.67± 80.58 (25.5- 393) nmol/l. There was no statistically significant difference between plasma pyridoxal phosphate levels of these two groups (P═0.430).

**Conclusion:**

Pyridoxine dependent epilepsy is under diagnosed because it is manifested by various types of seizures. Plasma pyridoxal phosphate levels did not differ in our patients with intractable or controlled epilepsy. If PDE is suspected on clinical basis, molecular investigation of ALDH7A1 mutations, as feasible test, until PDE biomarkers becomes available is recommended.

## Introduction

Seizure is a common neurologic problem. The incidence of epilepsy in children up to 15 yr old is reported 5-7 cases per 10000 (1).

Antiepileptic drugs (AEDs) as first modality of seizure treatment usually produce good seizure control in patients. At least 20% of children with seizure disorders on appropriate AEDs have recurrent seizures, intractable or uncontrolled epilepsies (2).

Pyridoxine dependency epilepsy (PDE) is one of the important and treatable causes of intractable epilepsies (3). In addition to PDE, some seizures in children may be controlled by administering pyridoxine as a antiepileptic drug (PRS) (4). In a study in IR.Iran, three of thirty infants and children with intractable epilepsy became seizure free with oral high dose of pyridoxine as adjunct to other AEDs (5). In a hospital based study in India, 7.4% of patients with intractable early onset cryptogenic epilepsy were diagnosed as definite cases of PDE (6).

Baxter classified PDE based on clinical criteria, control or recurrence of seizure after an experimental trial of pyridoxine administration and withdrawal to definite, probable and possible PDE (7). Due to different classic and atypical presentation of PDE and lack of specific, available biomarkers, only a few case reports are available, probably because of under diagnosing (8). 

Pyridoxal 5´-phosphate (PLP) is the major component of different pyridoxine vitamers in plasma (9). PLP is the main vitamer of B6 vitamin, which contributes as a cofactor in more than 100 enzyme-catalyzing reactions including different amino acids and amines in central nervous system (neurotransmitters) metabolic pathways (10). Low level of PLP in the brain results in neurological dysfunction, such as seizurs (11). Decreased level of PLP in cerebrospinal fluid (CSF) was seen in some cases of intractable epilepsy, PDE, pyridoxine phosphate oxidase deficiency and molybdenum cofactor deficiency (12). Low concentration PLP level in the brain can be presented with symptomatic neurological disorders. Low level of PLP in cerebrospinal fluid has been reported in intractable epilepsy previously (11, 12). Plasma PLP level is a measure to determine the adequacy of pyridoxine intake in human (13, 14). Decreased PLP level in CSF with normal plasma PLP level was reported in neonates with PDE (15). Despite this finding, study of Spanish patients with PDE showed some patients with PDE had low level of plasma PLP before pyridoxine treatment (16).

This study aimed to determine the value of plasma PLP level measurement in intractable and controlled epileptic patients.

## Material & Methods

This case control study was conducted in Pediatric Neurology Clinic of Mofid Children Hospital, Tehran, Iran from January 2010 to December 2010, a referral and teaching hospital. 

Patients with epilepsy after neonatal period up to 15 years old were included in the study. Exclusion criteria were 1- therapeutic vitamin B6 therapy during past six months, 2- intractable epilepsy due to a known symptomatic cause (neurocutaneous disease, brain structural anomalies or dysgenesis, brain tumor…).

This study was confirmed by the Ethics Committee of Shahid Beheshti University of Medical Sciences. Informed consent form was completed by parents.

Patients based on their seizures´ responses to antiepileptic drugs were classified as intractable or controlled epileptic cases. Children with seizures without therapeutic response to at least two appropriate antiepileptic drugs with optimum dose for more than two weeks were considered as intractable epileptic patients. Controlled epileptic patient was defined as a child whose seizures were controlled by maximum two antiepileptic drugs for at least six months. Thirty-three patients with refractory epilepsy were matched with 33 patients with controlled epilepsy as control group for age, sex, seizure type and family history of seizure.

Demographic, historical, physical examination, neuroimaging, electroencephalograms and laboratory data were recorded in a questionnaire by a child neurologist. Five millilitres blood was taken from each not fasting subject in vacutainers containing EDTA between 8 and 10 a.m. The samples were kept in crushed ice and protected from light. After centrifuge, plasma was frozen at -10 ˚C for future plasma PLP analyses.

Plasma PLP levels were measured using high-pressure liquid chromatography (HPLC) method with Younglin Instrument (South Korea). Intra-day coefficient of variation (CV) was less than 6% and inter-day (dayto-day) CV was less than 10%. Plasma pyridoxal 5’-phosphate deficiency was defined as PLP level less than 20 nmol/l (13). 

Categorical variables were analysed using Chi-square test. Continuous variables were analysed using t test or Mann Witney U test where appropriate.

## Results

Thirty-three patients with intractable epilepsy (10-162 months) and 33 patients with controlled epilepsy (14-173 months) were enrolled (Table 1). There was no statistically significant difference between two groups in terms of weight, parents consanguinity, daily multivitamin intake, seizure type (partial versus generalized). The differences for seizure age of onset, EEG, motor delay, cognition delay and speech delay between two groups were statistically significant. 

Mean plasma pyridoxal phosphate level (PLP) in patients with controlled epilepsy was 76.78±37.24 (nmol/l) (15.5-232.4). In patients with intractable epilepsy, mean plasma pyridoxal level was 98.67± 80.58 (25.5-393) nmol/l. There was no statistically significant difference between plasma pyridoxal phosphate levels of these two groups (P= 0.430).

Only one 38 months old male patient with controlled epilepsy on primidon therapy had plasma pyridoxal phosphate deficiency (PLP level: 15.5 nmol/l). His seizure onset was before 3 yr old. His neuroimaging, EEG, developmental indices were normal. He did not use daily multivitamin.

## Discussion

In this study, we did not find any significant statistical difference in plasma level of 5´- pyridoxal phosphate in patients with intractable or controlled epilepsy. Only one of our patients with controlled epilepsy on primidon therapy, without multivitamin intake, had plasma PLP level < 20 nmol/l. In a large population study, 25% of children < 13 yr old who did not use B6 supplement and 11% of same age group on vitamin B6 intake, had plasma PLP <20 nmol/l (14). Four of our patients had plasma PLP level < 30 nmol/l. They consisted of one male and one female patients with intractable and two male patients with controlled epilepsy. These patients did not use multivitamin daily, despite most of our patients who were on daily multivitamin intake, especially whom were on old AED therapy. Plasma PLP < 30 nmol/L has been reported in 25% of third grade elementary school children (8-9 yr old) in Indonesia. 

**Table 1:** Characteristics of the Study Subjects

	**Patients with** **controlled epilepsy**	**Patients with** **refractory epilepsy**	***P*** **-value**
**Age (months)**	84.6±44.2	85.6±45.8	0.959
**Female/male**	10/23	14/19	0.443
**Weight (kg)**	26.6±15.2	24.55±12.95	0.626
**Parents consanguinity**	13	20	0.139
**Seizure onset**	44.39 ± 37.65 2-136 mo	24.55±12.95 2-98 mo	0.003
**Partial seizure**	7	9	0.755
**Abnormal EEG**	17	26	0.038
**Speech delay**	2	12	0.003
**Cognition delay**	3	12	0.017
**Motor delay**	0	8	0.005
**Daily multivitamin**	3	8	0.185

These children did not get any drug or multivitamin (14). In this study, 2 patients with controlled epilepsy and 2 patients with intractable epilepsy had plasma PLP level less than 30 nmole/l. Pyridoxine treatments did not decrease number and frequency of seizures in these intractable epileptic patients, although they had been treated with high dose pyridoxine. These findings warrants study about adequacy of vitamin B6 intake in our children and importance of daily multivitamin intake in epileptic patients on AED therapy.

Classical PDE was described as prolonged, recurrent or intractable seizures in neonates and infants, without response to different AEDs but eventually intravenous pyridoxine administration control these seizures. 

PDE may present with atypical manifestation. Such presentations include: seizure onset after neonatal and infancy period (up to three years old), seizures that control by AEDs at first and later recurrence as intractable seizures, seizures that do not respond to pyridoxine immediately but finally will control later by it and longer interval between pyridoxine withdrawal and seizure recurrence (7,17).

Due to different seizure types and variable PDE manifestation, classical and atypical presentation, lack of pathognomonic electroencephalography or neuroimaging findings, an available, specific and noninvasive test will help to confirm it, in shorter time. 

Early diagnosis and treatment help to prevent or decreased adverse neurological outcomes of prolonged or intractable seizures. 

Such tests will eliminate need to experimental Baxter trial (pyridoxine stopping and waiting to seizure recurrence in patients whose intractable seizures were responded to pyridoxine) to prove PDE.

There are a few case reports during sixty years after PDE introduction in medical literature. Exact incidence of PDE in world is unknown. PDE incidence is reported from 1:20000 in a neonatal center study in Germany to about 1:700000 in United Kingdom (7, 18). This wide range partly is related to difference of two these study design. Studies have shown low PLP level in CSF of patients with PDE (12). PLP contributes as a cofactor in many neurotransmitters metabolisms in central venous system including gamma amino butyric acid (GABA) (10). Some studies tried to determine cut off value for PLP contents of CSF in different pediatric age groups (19- 20). Measurement of PLP level in CSF of patients with intractable epilepsy is a diagnostic test, but it is invasive and nonspecific. Decreased PLP concentration in CSF is seen in pyridoxine phosphate oxidase deficiency and molybdenum cofactor deficiency in addition to PDE.

In patients with PDE, cerebral PLP activity reduces through cerebral lysine catabolism. Increase in alpha aminoadipic semi aldehyde (α-AASA) concentration occurs due to alpha aminoadipic semi aldehyde (α-AASA) dehydrogenase or antiquitin (ATQ) enzyme deficiency. Delta 1piperideine-6-carboxylate (P6C) is cyclic form α-AASA. These two components are in a spontaneous equilibrium in central nervous system. Increased cerebral P6C concentration through knoevengal condensation, reduce cerebral PLP activity (21). Elevated level of pipecolic acid (PA), an intermediate product of lysine catabolism, in plasma and / or CSF was the first biomarker for PDE diagnosis (18). 

Increased PA concentration are seen in different conditions including liver diseases, peroxisomal disorders such Zellweger syndrome (22). Low CSF / plasma ratio of PA in these situations help to differentiate them from PDE (21). Increased urinary α-AASA excretion due to its accumulation in various body fluids (plasma, CSF, urine) has been introduced as a noninvasive, powerful and simple biomarker for PDE diagnosis, recently (23). 

Increased α-AASA urinary excretion is reported in other neurologic disorders including molybdenum cofactor and sulfite oxidase deficiencies (24). Increased urinary P6C excretion has been introduced as new biomarker, comparable with elevated urinary α-AASA excretion, in patients with PDE (25). Availability of these new biomarkers in some parts of the world is limited, because careful handling and proper freezing of urinary samples are essential to reliable interpretation of these tests (26, 27). Recently, Jung et al. suggested neonatal dried bloods spots for PDE screening (28). However, a sensitive biomarker as a screening tool for PDE is not available, yet.

Using molecular basis test, mutations of ALDH7A1 or antiquitin (ATQ) gene, in majority of patients with PDE have been reported. This gene is located on chromosome 5q31and encode α-AASA protein. ALDH7A1 mutations study was introduced for prenatal diagnosis of PDE at first (23). Today it is used to confirm PDE in patients who are suspected to have PDE on clinical basis in whom biomarker testing in not achievable or in patients with elevated urinary α-AASA or P6c and PA to establish PDE diagnosis, too (25).

**In conclusion, **Pyridoxine dependent epilepsy is under diagnosed because it is manifested by various types of seizures. Plasma pyridoxal phosphate levels did not differ in our patients with intractable or controlled epilepsy. If PDE is suspected on clinical basis, molecular investigation of ALDH7A1 mutations, as feasible test, until PDE biomarkers becomes available is recommended.

## Authors’ contribution

Pirzadeh Z: Contributions to the design of the work, the acquisition of data for the work, and drafting the work 

Ghofrani M: Contributions to the conception of the work, interpretation of data for the work, and revising the work critically for important intellectual content

Mollamohammadi M: Contributions to the acquisition of data for the work, analysis of data for the work, and drafting the work

All authors approved the final version to be published and agreed to be accountable for all aspects of the work in ensuring that questions related to the accuracy or integrity of any part of the work are appropriately investigated and resolved. 

## References

[1] Cown LD (2002). The epidemiology of the epilepsies in children. Ment Retard Dev Disabil Res Rev.

[2] French JA (2007). Refractory epilepsy: clinical overview. Epilepsia.

[3] Oliveira R, Pereira C, Rodrigues F, Alfaite C, Garcia P, Robalo C (2013). Pyridoxine-dependent epilepsy due to antiquitin deficiency: achieving a favourable outcome. Epileptic Disord.

[4] Baxter P (2001). Pyridoxine-dependent and pyridoxine-responsive seizures. Dev Med Child Neurol.

[5] Akhoondian J, Talebi S (2004). High dose oral pyridoxine for treatment of pediatric recurrent intractable seizure. MJIRI.

[6] Ramachandrannair R, Parameswaran M (2005). Prevalence of pyridoxine dependent seizures in south Indian children with early onset intractable epilepsy: A hospital based prospective study. Eur J Paediatr Neurol.

[7] Baxter P (1999). Epidemiology of pyridoxine dependent and pyridoxine responsive seizures in UK. Arch Dis Child.

[8] Yaghini O, Shahkarami MA, Shamsaii S (2010). Neglected atypical pyridoxine dependent seizures. Iran J Pediatr.

[9] Lumeng L, Lui A, Li TK (1980). Plasma content of B6 vitamers and its relationship to hepatic rat B6 metabolism. J Clin Inves.

[10] Clayton PT (2006). B6-responsive disorders: a model of vitamin dependency. J Inherit Metab Dis.

[11] Goyal M, Fequiere PR, McGrath TM, Hyland K (2013). Seizures with decreased levels of pyridoxal phosphate in cerebrospinal fluid. Pediatr Neurol.

[12] Footitt EJ, Heales SJ, Mills PB, Allen GF, Oppenheim M, Clayton PT (2011). Pyridoxal 5’-phosphate in cerebrospinal fluid; factors affecting concentration. J Inherit Metab Dis.

[13] Morris MS, Picciano MF, Jacques PF, Selhub J (2008). Plasma pyridoxal 5’-phosphate in the US population: the National Health and Nutrition Examination Survey, 2003-2004. Am J Clin Nutr.

[14] Setiawan B, Giraud DW, Driskell JA (2000). Vitamin B-6 inadequacy is prevalent in rural and urban Indonesian children. J Nutr.

[15] Shin YS, Rasshofer R, Endres W (1984). Pyridoxal-5’-phosphate concentration as marker for vitamin-B6-dependent seizures in the newborn. Lancet.

[16] Pérez B, Gutiérrez-Solana LG, Verdú A, Merinero B, Yuste-Checa P, Ruiz-Sala P (2013). Clinical, biochemical, and molecular studies in pyridoxine-dependent epilepsy Antisense therapy as possible new therapeutic option. Epilepsia.

[17] Gospe SM (2002). Pyridoxine-dependent seizures: findings from recent studies pose new questions. Pediatr Neurol.

[18] Plecko B, Hikel C, Korenke GC, Schmitt B, Baumgartner M, Baumeister F (2005). Pipecolic acid as a diagnostic marker of pyridoxine-dependent epilepsy. Neuropediatrics.

[19] Albersen M, Groenendaal F, van der Ham M, de Koning TJ, Bosma M, Visser WF (2012). Vitamin B6 vitamer concentrations in cerebrospinal fluid differ between preterm and term newborn infants. Pediatrics.

[20] Ormazabal A, Oppenheim M, Serrano M, García-Cazorla A, Campistol J, Ribes A (2008). Pyridoxal 5’-phosphate values in cerebrospinal fluid: reference values and diagnosis of PNPO deficiency in paediatric patients. Mol Genet Metab.

[21] Stockler S, Plecko B, Gospe SM Jr, Coulter-Mackie M, Connolly M, van Karnebeek C, Mercimek-Mahmutoglu S, Hartmann H, Scharer G, Struijs E, Tein I, Jakobs C, Clayton P, Van Hove JL (2011 ). Pyridoxine dependent epilepsy and antiquitin deficiency: clinical and molecular characteristics and recommendations for diagnosis, treatment and follow-up. Mol Genet Metab.

[22] Steinberg SJ, Dodt G, Raymond GV, Braverman NE, Moser AB, Moser HW (2006). Peroxisome biogenesis disorders. Biochim Biophys Acta.

[23] Mills PB, Struys E, Jakobs C, Plecko B, Baxter P, Baumgartner M (2006). Mutations in antiquitin in individuals with pyridoxine-dependent seizures. Nat Med.

[24] Struys EA, Nota B, Bakkali A, Al Shahwan S, Salomons GS, Tabarki B (2012). Pyridoxine-dependent epilepsy with elevated urinary α-amino adipic semialdehyde in molybdenum cofactor deficiency. Pediatrics.

[25] Struys EA, Bok LA, Emal D, Houterman S, Willemsen MA, Jakobs C (2012). The measurement of urinary Δ¹- piperideine-6-carboxylate, the alter ego of α-aminoadipic semialdehyde, in Antiquitin deficiency. J Inherit Metab Dis.

[26] Nam SH, Kwon MJ, Lee J, Lee CG, Yu HJ, Ki CS (2012). Clinical and genetic analysis of three Korean children with pyridoxine-dependent epilepsy. Ann Clin Lab Sci.

[27] Yang Z, Yang X, Wu Y, Wang J, Zhang Y, Xiong H (2014). Clinical diagnosis, treatment, and ALDH7A1 mutations in pyridoxine-dependent epilepsy in three Chinese infants. PLoS One.

[28] Jung S, Tran NT, Gospe SM Jr, Hahn SH (2013). Preliminary investigation of the use of newborn dried blood spots for screening pyridoxine-dependent epilepsy by LC-MS/MS. Mol Genet Metab.

